# Implementation Gap and Influencing Factors of Exercise Prescription for Older Adults: A Nationwide Survey in China

**DOI:** 10.1093/geroni/igaf122.1610

**Published:** 2025-12-31

**Authors:** Qin Zhang, Chengfan Qin, Nan Hua, Jing Chen

**Affiliations:** The First Affiliated Hospital, Zhejiang University School of Medicine, Hangzhou, Zhejiang, China (People’s Republic); The First Affiliated Hospital, Zhejiang University School of Medicine, Hangzhou, Zhejiang, China (People’s Republic); The First Affiliated Hospital, Zhejiang University School of Medicine, Hangzhou, Zhejiang, China (People’s Republic); The First Affiliated Hospital, Zhejiang University School of Medicine, Hangzhou, Zhejiang, China (People’s Republic)

## Abstract

**Objective:**

This study aimed to assess the knowledge, attitudes, and practices of exercise prescription among healthcare professionals in China, and to examine factors influencing exercise prescription for older adults.

**Methods:**

A nationwide survey was conducted to recruit healthcare professionals in geriatric care in China. Descriptive statistics summarized their knowledge, attitudes, practices, and barriers to implementation. Thematic analysis was used to analyze participants’ insights and suggestions. Ordinal logistic regression examined factors associated with prescription practices, and mediation analysis tested whether knowledge and attitudes mediated the effect of training on practice.

**Results:**

A total of 1,890 participants were included. Most doctors and nurses (43%–59%) reported a slight awareness of exercise prescriptions. Over half had never received training, yet nearly 95% rated prescribing exercise for older adults as quite to very important. Written prescriptions were uncommon across tertiary hospitals (9%), non-tertiary hospitals (4%), and primary healthcare institutions (10%). The perceived main barriers were time constraints, lack of knowledge, safety concerns, and insufficient family support. Trained doctors (OR = 4.26, 95% CI: 3.36–5.64, p < 0.001) and nurses (OR = 2.52, 95% CI: 1.91–3.32, p < 0.001) were more likely to provide written prescriptions. Knowledge and attitudes mediated the relationship between training and prescription practices.

**Conclusions:**

Chinese healthcare professionals had limited knowledge of exercise prescriptions, with low usage of written prescriptions. They recognized the importance of exercise prescriptions and maintained a positive attitude. Knowledge and attitudes mediated the impact of training on prescription practices.

